# Innate cell profiles during the acute and convalescent phase of SARS-CoV-2 infection in children

**DOI:** 10.1038/s41467-021-21414-x

**Published:** 2021-02-17

**Authors:** Melanie R. Neeland, Samantha Bannister, Vanessa Clifford, Kate Dohle, Kim Mulholland, Philip Sutton, Nigel Curtis, Andrew C. Steer, David P. Burgner, Nigel W. Crawford, Shidan Tosif, Richard Saffery

**Affiliations:** 1 Infection and Immunity Theme, Murdoch Children’s Research Institute, Parkville, VIC Australia; 2 Department of Paediatrics, The University of Melbourne, Parkville, VIC Australia; 3 Infectious Diseases Unit, The Royal Children’s Hospital, Parkville, VIC Australia; 4 Laboratory Services, The Royal Children’s Hospital, Parkville, VIC Australia; 5 Department of General Medicine, The Royal Children’s Hospital, Parkville, VIC Australia

**Keywords:** Infection, Infectious diseases, Innate immunity, SARS-CoV-2

## Abstract

Children have mild severe acute respiratory syndrome coronavirus 2 (SARS-CoV-2) confirmed disease (COVID-19) compared to adults and the immunological mechanisms underlying this difference remain unclear. Here, we report acute and convalescent innate immune responses in 48 children and 70 adults infected with, or exposed to, SARS-CoV-2. We find clinically mild SARS-CoV-2 infection in children is characterised by reduced circulating subsets of monocytes (classical, intermediate, non-classical), dendritic cells and natural killer cells during the acute phase. In contrast, SARS-CoV-2-infected adults show reduced proportions of non-classical monocytes only. We also observe increased proportions of CD63+ activated neutrophils during the acute phase to SARS-CoV-2 in infected children. Children and adults exposed to SARS-CoV-2 but negative on PCR testing display increased proportions of low-density neutrophils that we observe up to 7 weeks post exposure. This study characterises the innate immune response during SARS-CoV-2 infection and household exposure in children.

## Abstract (web summary)

Childhood infection with SARS CoV2 is associated with a milder course of infection but the immunopathogenesis of this remains unclear. Here the authors explore immunological differences in the innate immune system during acute and convalescent SARS CoV2 infection in the young.

## Introduction

Children have mild severe acute respiratory syndrome coronavirus 2 (SARS-CoV-2) confirmed disease (COVID-19) compared to adults, and up to one-third are asymptomatic^1–3^. Recent work suggests that children are also less likely to become infected with SARS-CoV-2, which is strikingly different to the higher prevalence and severity observed in children for most other respiratory viruses^4^. The immunological mechanisms underlying protection in younger age groups remain unclear.

Immune profiling studies of severe paediatric COVID-19 and multisystem inflammatory syndrome in children (MIS-C) have suggested that widespread inflammation and dynamic changes in T cell immunity may play a key role in disease pathogenesis^5,6^. However, data on the cellular immune response in non-hospitalised children with mild infection, the most common clinical course of children with COVID-19, are limited. A previous study of two parents with PCR-confirmed symptomatic SARS-CoV-2 infection and their three SARS-CoV-2 PCR-negative children showed that all exposed children mounted a cellular immune response characterised by striking changes in the frequency of innate immune cells over time^7^, suggesting that household exposure to SARS-CoV-2 induces a change in the immune response even in the absence of virological confirmation of infection.

Here, we compare the innate immune cell profiles of children and adults with or exposed to SARS-CoV-2, revealing that infection in children is characterised by increased activation of neutrophils and reduced proportions of circulating myeloid cells, and that household exposure to SARS-CoV-2 induces the generation of low-density neutrophils that can be observed up to 7 weeks post exposure in both children and adults.

## Results and discussion

Participants were families presenting for SARS-CoV-2 testing at the Royal Children’s Hospital Melbourne, Australia, between April and August 2020. Acute samples were collected at the earliest timepoint from the first positive SARS-CoV-2 PCR test result and convalescent samples were collected 4–7 weeks following (Table 1). We used multi-parameter flow cytometry and unsupervised analysis to investigate the innate immune response in blood samples collected in the acute or convalescent phase from children and adults infected with or exposed to SARS-CoV-2. SARS-CoV-2^-^positive individuals were non-hospitalised patients who had a positive PCR test for SARS-CoV-2 on nasopharyngeal swab and experienced mild symptoms (Table 1), including coryza, headaches, nausea, fever, cough, sore throat, malaise, headaches and muscle aches (see supplemental data for individual symptom data). SARS-CoV-2-exposed individuals were defined as those who had exposure to SARS-CoV-2 in the household with a positive household close contact but were SARS-CoV-2 PCR negative on repeated nasopharyngeal swabs. All participants in the exposed group had up to five repeat SARS-CoV-2 PCR tests at 5-7 day intervals for 4 weeks. All repeat tests remained negative for all participants in this group. Timing of sampling in exposed individuals in relation to positive PCR test in their household close contacts is listed in Table 1. SARS-CoV-2 PCR testing was done at the Royal Children’s Hospital Microbiology laboratory according to the protocols described in the methods using the LightMix® Modular SARS and Wuhan CoV E-gene kit (TIB Molbiol, Berlin, Germany) and the AusDiagnostics Respiratory Pathogens 16-well assay (Ausdiagnostics, Mascot, Australia).

**Table 1:** Demographics of study cohort.

	CHILDREN				ADULTS			
	SARS-CoV-2 positive		SARS-CoV-2 exposed		SARS-CoV-2 positive		SARS-CoV-2 exposed	
	Acute	Convalescent	Acute	Convalescent	Acute	Convalescent	Acute	Convalescent
Number	11	16	7	14	17	8	21	24
Age (years), median (min-max)	4.45 (1–14)	10 (1–17)	9 (3–17)	9 (3–17)	35 (19–62)	40 (20–62)	38 (21–50)	41 (22–56)
Time of sampling since test (days), median (min-max)	8 (2–13)	36 (30–49)	1 (0–12)	37 (28–37)	11.5 (4–18)	41 (26–50)	4 (0–15)	31 (23–65)
Time of sampling since positive PCR test in contacts			2 (2–15)	39 (30–41)			7 (2–18)	35 (28–65)

**Fig. 1:**
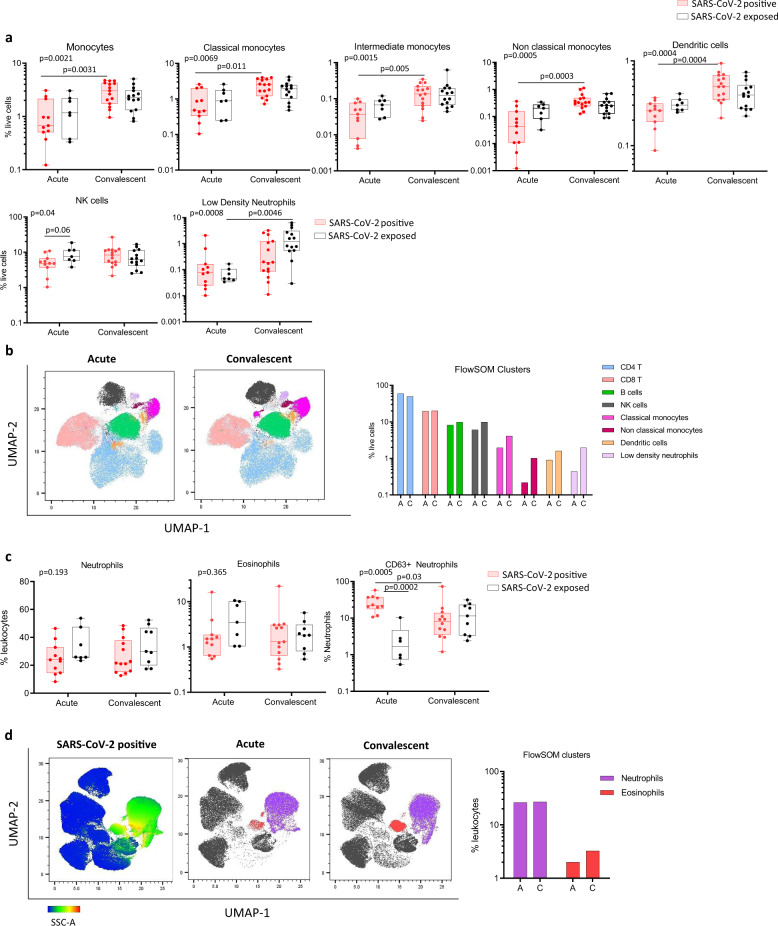
Innate cell profiles of children infected with or exposed to SARS-CoV-2 during the acute and convalescent phase. **a** Frequency of monocytes and subsets (classical, non-classical, intermediate), dendritic cells, NK cells and low-density immature neutrophils in PBMC samples of children during infection (SARS-CoV-2 positive *n* = 11, SARS-CoV-2 exposed n = 7) and in convalescence (SARS-CoV-2 positive *n* = 16, SARS-CoV-2 exposed *n* = 14). **b** Hierarchical clustering was performed on PBMC samples using FlowSOM and projected onto a UMAP plot. Frequencies of clusters corresponding to CD4 T, CD8 T, B cells, NK cells, classical monocytes, non-classical monocytes, dendritic cells and low-density neutrophils during acute (A) and convalescent (C) phase are reported. **c** Frequency of neutrophils and eosinophils in fresh whole blood (SARS-CoV-2 positive *n* = 11, SARS-CoV-2 exposed *n* = 7) and in convalescence (SARS-CoV-2 positive *n* = 13, SARS-CoV-2 exposed *n* = 9). Neutrophils were assessed for expression of activation marker CD63 (SARS-CoV-2 positive *n* = 11, SARS-CoV-2 exposed *n* = 6) and in convalescence (SARS-CoV-2 positive *n* = 13, SARS-CoV-2 exposed *n* = 9). **d** Hierarchical clustering was done on whole blood samples using FlowSOM and projected onto a UMAP plot, coloured by intensity of SSC-A expression in the first plot, and then by annotated granulocyte cell population. Frequencies of clusters corresponding to neutrophils and eosinophils are reported. *P* values by Kruskal-Wallis rank sum test and Dunn’s multiple comparison testing. All statistical tests were performed two-sided. Boxplots show the medians, the 1st and 3rd quartile as well as the smallest and largest values as whiskers.

Low circulating numbers of non-classical monocytes are an emerging characteristic feature of SARS-CoV-2 infection in adults^8,9^. Here, we observed low proportions of total monocytes in children with SARS-CoV-2 infection during the acute phase that were restored in convalescence (median 0.67% vs 3.0% of PBMCs, *p* = 0.0031, Fig. 1a). This was evident in all three circulating monocyte subsets of infected children, with the most dramatic changes observed in the intermediate (CD14^+^CD16^+^) and non-classical (CD14^low^CD16^+^) subsets between acute and convalescent phases: intermediate monocytes (median 0.07% vs 0.14%, *p* = 0.005) and non-classical monocytes (median 0.04% vs 0.33%, *p* = 0.0003, Fig. 1a). This effect was also observed in SARS-CoV-2 infected adults for non-classical monocytes at baseline and convalescence (median 0.19% vs 1.1%, *p* = 0.01, Supplementary Fig. 1a), however no differences were observed in adults for the other two monocyte subsets. Children also showed a reduction in dendritic cells during the acute phase of SARS-CoV-2 infection that were restored in convalescence (median 0.26% vs 0.50%, *p* = 0.0004, Fig. 1a). No differences were observed in adults for the dendritic cell population (Supplementary Fig. 1a).

Natural killer (NK) cells, known to play a key role in bridging the innate and adaptive immune response against viral infections^10^, have been scarcely investigated in paediatric COVID-19. Here, we found that SARS-CoV-2 positive children had reduced proportions of NK cells during the acute phase compared with SARS-CoV-2-exposed children (median 4.8% vs 8.3% of PBMC, *p* = 0.06, Fig. 1a). This difference was not observed in adults infected with or exposed to SARS-CoV-2 (median 9.1% vs 8.8%, respectively, *p* = 0.99, Supplementary Fig. 1a).

To confirm the findings observed by manual gating, we performed unsupervised clustering analysis of PBMC samples from acute and convalescent samples of SARS-CoV-2-infected children using FlowSOM^11^. To visualise these data in two dimensions, the non-linear dimensionality reduction technique UMAP^12^ was applied and the cells colour highlighted by their respective FlowSOM cluster (Fig. 1b). Based on surface marker expression, the clusters were classified into CD4 T cells, CD8 T cells, B cells, NK cells, classical monocytes, non-classical monocytes, dendritic cells and low-density neutrophils. The frequency of each cluster during the acute and convalescent phase revealed identical reductions in NK cells, monocytes and dendritic cells to those observed by manual gating in SARS-CoV-2 infected children (Fig. 1b).

The observation that several innate cell populations were considerably reduced in the circulation during paediatric infection suggests that these cells may be recruited to sites of infection. In a study in adults with moderate or severe COVID-19, there was an increase in dendritic cells and NK cells in the bronchoalveolar lavage of patients with mild COVID-19, whilst severe disease was characterised by increases in monocyte-derived macrophages^13^. Another study compared the blood and lung profiles of patients with severe COVID-19, revealing that non-classical monocytes preferentially migrate from the blood into the lungs during disease^14^.

We observed further differences in the myeloid compartment in our whole blood analysis, particularly in SARS-CoV-2 infected children. In this work, we focused on defining the phenotype and frequency of circulating granulocytes. While total proportions of neutrophils and eosinophils were not significantly different between groups in children or adults (Fig. 1c–d, Supplementary Fig. 1b), an increase in the proportion of CD63^+^ neutrophils was observed in SARS-CoV-2 infected children relative to SARS-CoV-2-exposed children in the acute phase (median 22% vs 1.7%, *p* = 0.0002, Fig. 1c). The proportion of CD63^+^ neutrophils in SARS-CoV-2 infected children decreased from 22% in the acute phase to 8.1% in convalescence (*p* = 0.03, Fig. 1c). CD63 has been shown to be upregulated on the surface of neutrophils after activation and involved in the release of pro-inflammatory mediators as part of the anti-viral immune response^15^. This response was not observed in SARS-CoV-2-infected adults (Supplementary Fig. 1b), warranting further investigation of this activated neutrophil subtype in mediating the acute phase immune response in children. Other markers of innate cell activation including HLADR, CD11b and CD11c were not different between groups.

The convalescent time point in both adults and children exposed to SARS-CoV-2 was associated with the appearance of low-density immature neutrophils (SSC^hi^CD16^+^) in the PBMC fraction, identified by manual gating (Fig. 1a) and replicated in unsupervised analyses (Fig. 1b). Pre- and immature-neutrophils in PBMC fractions have been recently described in adults with severe COVID-19^16^. Other studies have shown increases in low density or immature neutrophils in both mild and severe disease that are positively correlated with systemic inflammatory cytokine levels^17,18^. In our study, the generation of low-density immature neutrophils were observed only in SARS-CoV-2 negative patients several weeks following exposure, suggesting they may also play a role in protective immune responses.

This work provides a comprehensive characterisation of the innate cells responding during the acute and convalescent phase of mild paediatric COVID-19. Further investigations comparing these responses using longitudinally collected samples from the same paediatric patients throughout infection and long-term recovery, as well as in-vitro functional responses of these cells to SARS-CoV-2, will provide additional insight. We compared the innate immune responses of children and adults with mild SARS-CoV-2 infection, revealing that infection in children is characterised by increased activation of neutrophils and low circulating proportions of all monocyte subsets, dendritic cells and natural killer cells, in contrast to SARS-CoV-2-infected adults who showed reductions in the non-classical monocyte fraction only. We also show that exposure to SARS-CoV-2 induces changes in the immune response in the absence of virological confirmation of infection, highlighted by the emergence of low-density immature neutrophils up to 7 weeks post exposure in both children and adults. These findings provide insights into the immune mechanisms that may contribute to age-related differences in COVID-19.

## Methods

### Patients and diagnosis of SARS-CoV-2 infection

Clinical characteristics and demographics of the patients in this study are described in Table 1. Patients were recruited through the Royal Children’s Hospital Respiratory Infection Clinic between April-August 2020. Acute samples were collected within 2 weeks of the first test result and convalescent samples were collected 4–7 weeks following test results. SARS-CoV-2 positive individuals were SARS-CoV-2 PCR positive on nasopharyngeal swabs and experienced mild symptoms. SARS-CoV-2 exposed individuals were SARS-CoV-2 PCR negative on repeated nasopharyngeal swabs and close contacts of confirmed SARS-CoV-2 positive patients in their households. Close contacts were defined as having face-to-face contact for more than 15 min and shared a closed space with a confirmed case of COVID-19, according to state guidelines. All clinical information was recorded in REDCap V10.1.2.

Combined oropharyngeal and nasopharyngeal (or deep nasal) swabs were collected according to national guidelines using dry FLOQSwabs® (Copan, Brescia, Italy). Briefly, FLOQSwabs were eluted in 500 μL of phosphate buffered saline (PBS). Nucleic acid extraction was performed using 200 μL eluent, extracted using the Roche Magnapure 96 extraction system (Roche, Basel, Switzerland), according to the manufacturer’s instructions. The majority of SARS-CoV-2 samples were first tested using the LightMix® Modular SARS and Wuhan CoV E-gene kit (TIB Molbiol, Berlin, Germany) using 10 μL nucleic acid extract, according to the manufacturer’s instructions. RT-PCR was performed on the LightCycler 480 II Real-Time PCR System (Roche). Some patient samples were initially tested for SARS-CoV-2 using the AusDiagnostics Respiratory Pathogens 16-well assay (Mascot, Australia), on the Ausdiagnostics High-Plex 24 System, using 10 μL nucleic acid extract. Where possible, samples that were positive on a screening assay with a single gene target were confirmed by testing on a second assay.

Both the TIB Molbiol (targeting e-gene) and the AusDiagnostics respiratory multiplex tandem PCR (targeting ORF-1 gene) are commercially available assays. A published evaluation estimated the Tib Molbiol e-gene assay to have a sensitivity of 96.5% and specificity of 98.5%^19^. The Ausdiagnostics assay has been independently compared with the results of the Victorian Infectious Diseases Reference Laboratory, with 98.4% positive agreement between the Ausdiagnostics assay and the reference assay^20^.

Where available, CT scores at diagnosis for SARS-CoV-2 positive patients are provided in the Supplemental data. A secondary analysis excluding children with weakly positive SARS-CoV-2 PCR results (CT > 30) is presented in Supplementary Fig. 2. As co-infection between SARS-CoV-2 and other respiratory viruses has been reported, we also performed testing for other common respiratory viruses. In our acute-phase and convalescent-phase SARS-CoV-2 positive cohorts, only 1 child in each group (0 adults) tested positive for other respiratory viruses (enterovirus, adenovirus) at the time of SARS-CoV-2 testing (see Supplemental data).

### Flow cytometry of PBMC and whole blood

Blood was collected in EDTA tubes from each participant. Immediately following collection, 100 µl of whole blood was aliquoted for flow cytometry analysis. The remaining EDTA blood samples were processed into PBMC^21^. For flow cytometry analysis of whole blood samples, whole blood was lysed with 1 mL of red cell lysis buffer for 10 min at room temperature. Cells were washed with 1 mL PBS and centrifuged at 350 × *g* for 5 min. Following two more washes, cells were resuspended in PBS for viability staining using near infra-red viability dye according to manufacturers instructions. For flow cytometry analysis of freshly isolated PBMC, cells were washed in 1 mL PBS prior to viability staining using BV510 viability dye according to manufacturers instructions. For both whole blood and PBMC samples, the viability dye reaction was stopped by the addition of FACS buffer (2% heat-inactivated FCS in 2 mM EDTA) and cells were centrifuged at 350 × *g* for 5 min. Cells were then resuspended in human FC-block according to manufacturers instructions for 5 min at room temperature. The whole blood or PBMC antibody cocktails (Supplementary Table 1) made up at 2X concentration were added 1:1 with the cells and incubated for 30 min on ice. Following staining, cells were washed with 2 mL FACS buffer and centrifuged at 350 × *g* for 5 min. Cells were then resuspended in 2% PFA for a 20 min fixation on ice, washed, and resuspended in 150 µl FACS buffer for acquisition using the BD LSR X-20 Fortessa and BD FACS DIVA V 9.0 software. For all flow cytometry experiments, compensation was done at the time of sample acquisition using compensation beads. Supplementary Figure 3 depicts the manual gating strategy for PBMC and whole blood samples.

### Data analysis

Results were analysed (manual gating, FlowSOM, UMAP) using FlowJo Version 10.7.1 software. FlowSOM and UMAP analyses was conducted using concatenated files containing 10,000 randomly selected live single cells per sample. FlowSOM and UMAP analyses of PBMC were conducted on a concatenated file containing 270,000 events from SARS-CoV-2 positive child samples collected during acute or convalescent phase. FlowSOM and UMAP analyses of whole blood were conducted on a concatenated file containing 216,000 events from SARS-CoV-2 positive and exposed child samples collected during acute or convalescent phase (8000 randomly selected live single cells per sample). Manually gated results are presented as proportion of live cells (for PBMC) or as proportion of CD45^+^ leucocytes (for whole blood). Data was plotted in Prism version 8.0.0. To perform the differential abundance analysis for all groups, the Kruskal-Wallis rank sum test was used, with subsequent Dunn’s multiple comparison testing. All statistical analysis was performed in Prism version 8.0.0. Boxplots show the medians, the 1st and 3rd quartile as well as the smallest and largest values as whiskers. Individual data points are shown.

### Ethics

This project received ethical approval from The Royal Children’s Hospital Melbourne Human Research Ethics Committee (HREC): HREC/63666/RCHM-2019. All donors or their legal guardians provided written informed consent.

### Reporting summary

Further information on research design is available in the Nature Research Reporting Summary linked to this article.

## Supplementary information

Supplementary Information

Description of Additional Supplementary Files

Supplementary Data 1

Reporting summary

## Data Availability

The authors declare that the data supporting the findings of this study are available within the paper and its supplementary information files. Source data are provided with this paper.

## Source data

Source Data
