# Supplementary material for: Innate cell profiles during the acute and convalescent phase of SARS-CoV-2 infection in children
Source: Nat Commun. 2021 Feb 17;12:1084. doi: 10.1038/s41467-021-21414-x (PMC7889848; doi:10.1038/s41467-021-21414-x)
Supplement: Supplementary file 1 — Supplementary Information [file 41467_2021_21414_MOESM1_ESM.pdf]

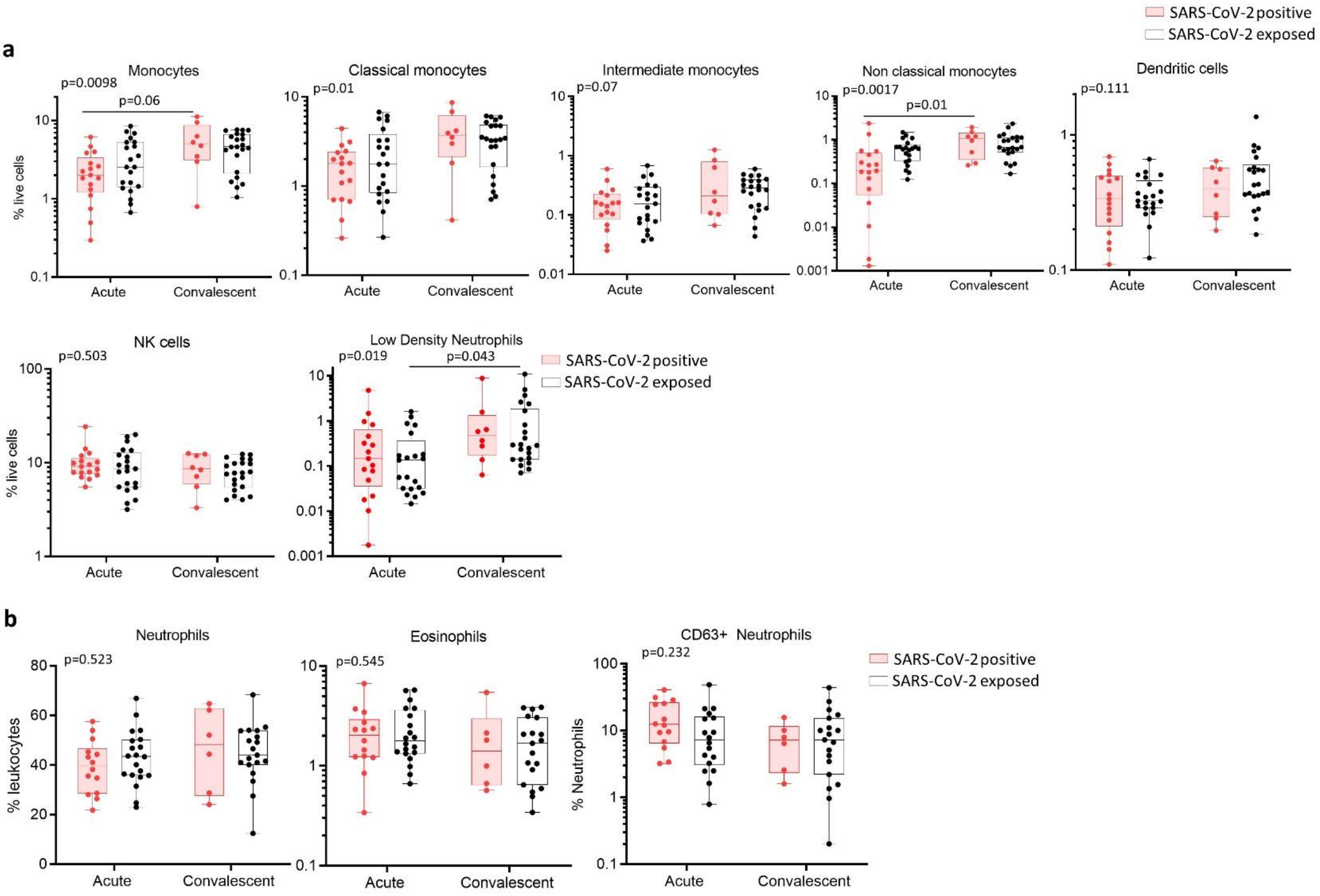

Supplementary Figure 1. Innate cell profiles of adults infected with or exposed to SARS-CoV-2 during the acute and convalescent phase. (a) Frequency of monocytes and subsets (classical, non-classical, intermediate), dendritic cells, NK cells and low-density immature neutrophils in PBMC samples of adults during infection (SARS-CoV-2 positive n=17, SARS-CoV-2 exposed n=21) and in convalescence (SARS-CoV-2 positive n=8, SARS-CoV-2 exposed n=22). **(b)** Frequency of neutrophils and eosinophils in fresh whole blood samples of adults during infection (SARS-CoV-2 positive n=14, SARS-CoV-2 exposed n=20) and in convalescence (SARS-CoV-2 positive n=6, SARS-CoV-2 exposed n=19). P values by Kruskal-Wallis rank sum test and Dunn's multiple comparison testing. All statistical tests were performed two-sided. Boxplots show the medians, the 1st and 3rd quartile as well as the smallest and largest values as whiskers.

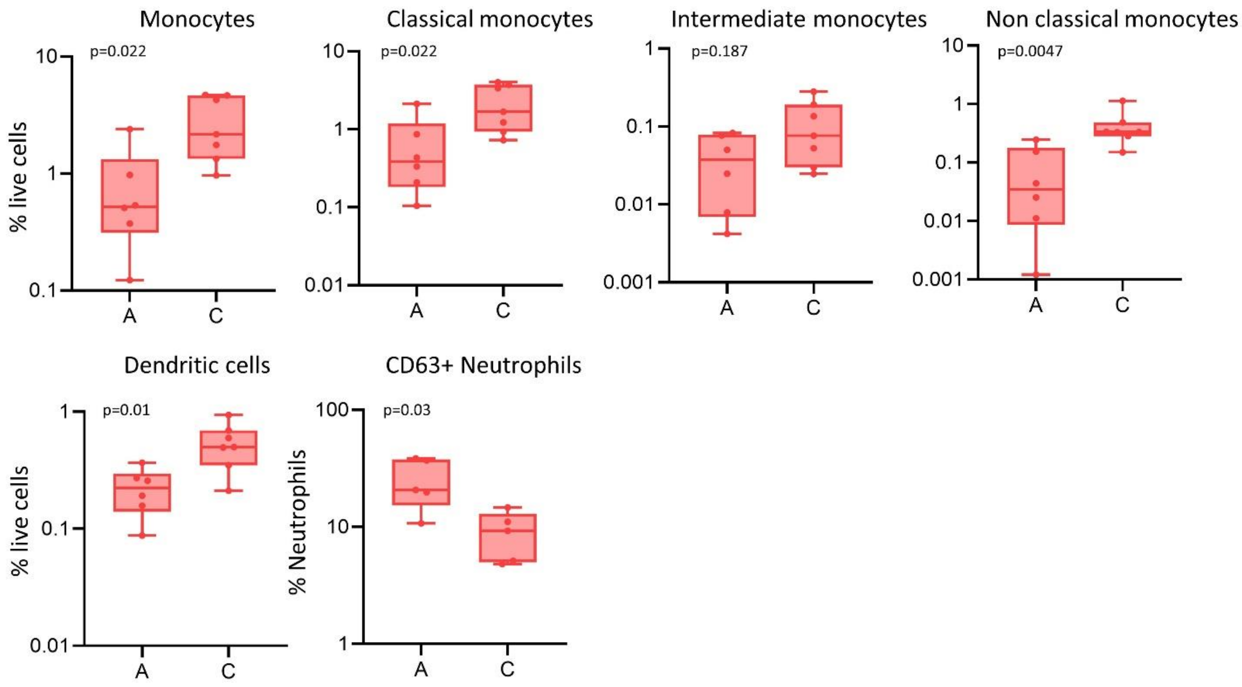

Supplementary Figure 2. Innate cell profiles of children with SARS-CoV-2 during the acute (A) and convalescent (C) phase, excluding children with weak positive PCR results (CT>30). Frequency of monocytes and subsets (classical, non-classical, intermediate), dendritic cells and CD63+ neutrophils in samples from SARS-CoV-2 positive children with initial CT<30 (samples collected during acute phase (n=6) and samples collected during convalescence (n=7)). P values by two-sided Mann-Whitney U test. Boxplots show the medians, the 1st and 3rd quartile as well as the smallest and largest values as whiskers**.**

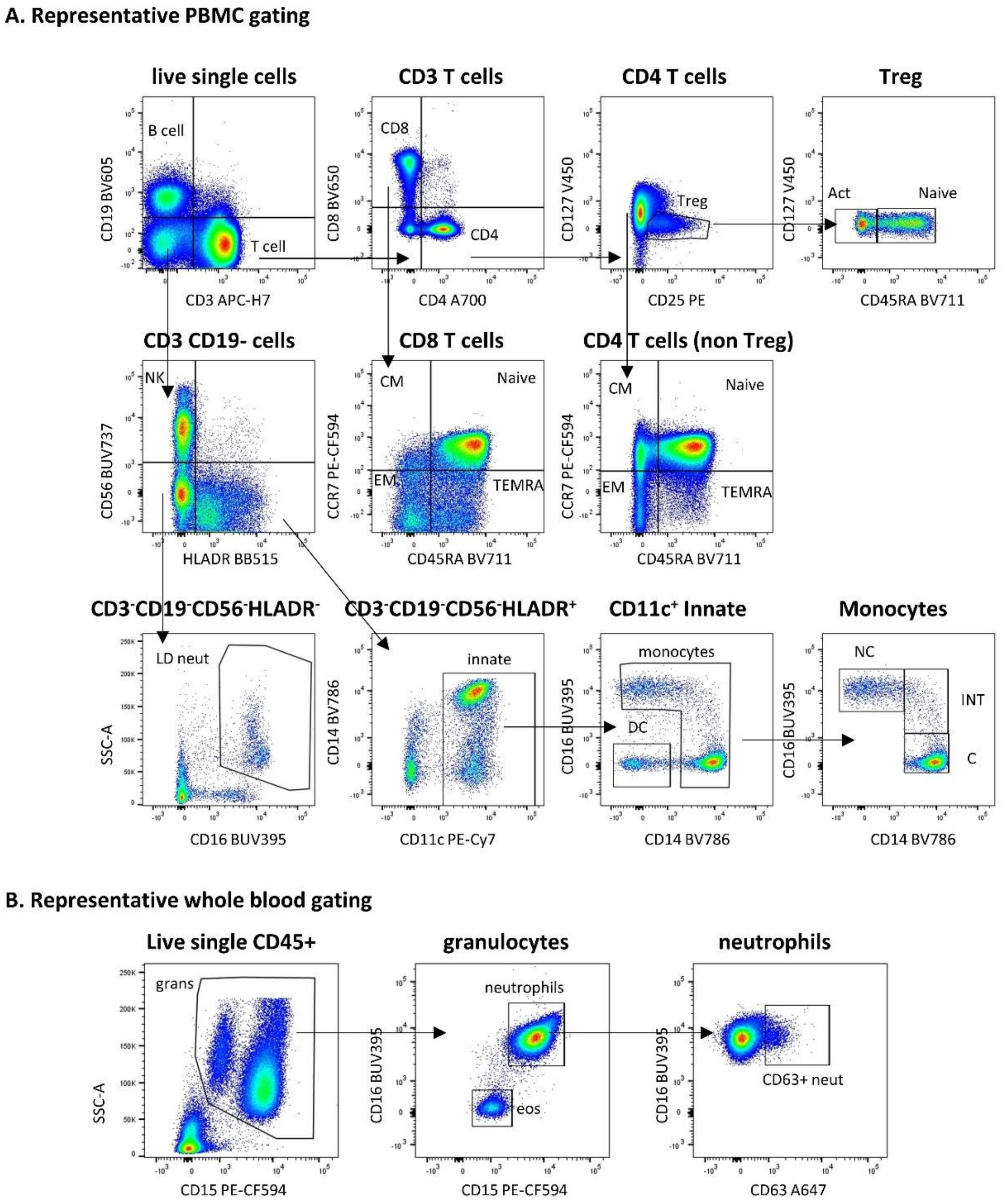

Supplementary Figure 3. Representative flow cytometry gating strategies for PBMC and whole blood. (A) Within the PBMC fraction, B cells were selected based on CD19 expression, and the total T cell fraction based on CD3 expression. CD4 and CD8 T cells, and their naïve, effector, memory and regulatory (Treg) subsets were also quantified. CD3$^-$CD19$^-$ cells were classified into NK cells (CD56$^+$) and innate cells (HLA-DR$^+$). Within the innate cell fraction, CD14$^+$ monocytes and CD11c$^+$ DCs were identified. Monocyte subsets were identified based on CD16 expression and classified into classical, intermediate, and non-classical subsets. Low density neutrophils were observed in the PBMC fraction at convalescence, characterised by a high SSC profile and CD16 expression. **(B)** For whole blood, granulocytes were selected within CD45$^+$ leukocytes based on their SSC profile and CD15 expression. Neutrophils were CD15$^+$CD16$^+$ and eosinophils were CD15$^+$CD16$^-$. CD63 expression was investigated within the neutrophil population.

Supplementary Table 1. Flow cytometry antibody cocktails

Surface Marker	Fluorophore	Clone	Final Dilution
Whole blood panel			
CD14	BV786	M5E2	1:50
CD11b	BUV805	ICRF44	1:100
CD45	BV711	HI30	1:100
CD56	BUV737	NCAM16.2	1:100
CD11c	PE-Cy7	B-ly6	1:100
CD63	A647	H5C6	1:100
CD4	A700	RPA-T4	1:100
CD3	BB515	UCHTI	1:100
PD1	BB700	EH12.1	1:100
CD15	PE-CF594	W6D3	1:200
HLADR	V500	G46-6	1:200
CD19	BV605	SJ25C1	1:200
CD8	BV650	RPA-T8	1:200
CD16	BUV395	3G8	1:400
Live/dead	N-IR		
PBMC panel			
CD25	PE	M-A251	1:25
CD127	V450	HIL7RM21	1:50
CD3	APCH7	SK7	1:50
CD14	BV786	M5E2	1:50
CD45RA	BV711	HI100	1:100
HLADR	BB515	G46-6	1:100
CD56	BUV737	NCAM16.2	1:100
CD11c	PE-Cy7	B-ly6	1:100
CD4	A700	RPA-T4	1:100
PD1	BB700	EH12.1	1:100
CCR7	PE-CF594	150503	1:200
CD19	BV605	SJ25C1	1:200
CD8	BV650	RPA-T8	1:200
CD16	BUV395	3G8	1:400
Live/dead	BV510		
