# Supplementary material for: Innate cell profiles during the acute and convalescent phase of SARS-CoV-2 infection in children
Source: Nat Commun. 2021 Feb 17;12:1084. doi: 10.1038/s41467-021-21414-x (PMC7889848; doi:10.1038/s41467-021-21414-x)
Supplement: Supplementary file 2 — Description of Additional Supplementary Files [file 41467_2021_21414_MOESM2_ESM.pdf]

Description of Additional Supplementary Files

Supplementary Data 1. Clinical details of included participants, including SARS-CoV-2 PCR CT scores, SARS-CoV-2 serology results, symptom data and other respiratory viral testing results.
