# Supplementary material for: Innate cell profiles during the acute and convalescent phase of SARS-CoV-2 infection in children
Source: Nat Commun. 2021 Feb 17;12:1084. doi: 10.1038/s41467-021-21414-x (PMC7889848; doi:10.1038/s41467-021-21414-x)
Supplement: Supplementary file 4 — Reporting summary [file 41467_2021_21414_MOESM4_ESM.pdf]

nature research

Corresponding author(s): Melanie Neeland

Last updated by author(s): Jan 12, 2021

Reporting Summary

Nature Research wishes to improve the reproducibility of the work that we publish. This form provides structure for consistency and transparency in reporting. For further information on Nature Research policies, see our Editorial Policies and the Editorial Policy Checklist.

Statistics

For all statistical analyses, confirm that the following items are present in the figure legend, table legend, main text, or Methods section.

n/a	Confirmed	
☐	☒	The exact sample size (*n*) for each experimental group/condition, given as a discrete number and unit of measurement
☐	☒	A statement on whether measurements were taken from distinct samples or whether the same sample was measured repeatedly
☐	☒	The statistical test(s) used AND whether they are one- or two-sided<br>*Only common tests should be described solely by name; describe more complex techniques in the Methods section.*
☒	☐	A description of all covariates tested
☐	☒	A description of any assumptions or corrections, such as tests of normality and adjustment for multiple comparisons
☒	☐	A full description of the statistical parameters including central tendency (e.g. means) or other basic estimates (e.g. regression coefficient) AND variation (e.g. standard deviation) or associated estimates of uncertainty (e.g. confidence intervals)
☐	☒	For null hypothesis testing, the test statistic (e.g. *F*, *t*, *r*) with confidence intervals, effect sizes, degrees of freedom and *P* value noted<br>*Give P values as exact values whenever suitable.*
☒	☐	For Bayesian analysis, information on the choice of priors and Markov chain Monte Carlo settings
☒	☐	For hierarchical and complex designs, identification of the appropriate level for tests and full reporting of outcomes
☒	☐	Estimates of effect sizes (e.g. Cohen's *d*, Pearson's *r*), indicating how they were calculated

Our web collection on statistics for biologists contains articles on many of the points above.

Software and code

Policy information about availability of computer code

Data collection	REDCap V10.1.2, BD FACS DIVA V9.0
Data analysis	Flowjo V10.7.1 (including UMAP and FlowSOM plugins within Flowjo V10.7.1), GraphPad Prism V8,

For manuscripts utilizing custom algorithms or software that are central to the research but not yet described in published literature, software must be made available to editors and reviewers. We strongly encourage code deposition in a community repository (e.g. GitHub). See the Nature Research guidelines for submitting code & software for further information.

Data

Policy information about availability of data

All manuscripts must include a data availability statement. This statement should provide the following information, where applicable:

- Accession codes, unique identifiers, or web links for publicly available datasets
- A list of figures that have associated raw data
- A description of any restrictions on data availability

The authors declare that the data supporting the findings of this study are available within the paper and its supplementary information files. Source data are provided with this paper.

Field-specific reporting

Life sciences study design

All studies must disclose on these points even when the disclosure is negative.

Sample size	Blood samples from children (total 48); 27 SARS-CoV-2 positive (11 acute, 16 convalescent) and 21 SARS-CoV-2 exposed (7 acute, 14 convalescent). Blood samples from adults (total 70): 25 SARS-CoV-2 positive (17 acute, 8 convalescent) and 45 SARS-CoV-2 exposed (21 acute, 24 convalescent). All participants recruited into our study who donated blood between April-August 2020 were included in this manuscript.
Data exclusions	No data were excluded from the analysis
Replication	Findings cannot be replicated in these exact individuals as all available cells were used for the experiments conducted within this manuscript. However, results were confirmed by two separate analyses (manual and unsupervised approaches).
Randomization	Samples were randomized prior to data analysis.
Blinding	Investigators were blinded to COVID-19 status during data collection and analysis.

Reporting for specific materials, systems and methods

We require information from authors about some types of materials, experimental systems and methods used in many studies. Here, indicate whether each material, system or method listed is relevant to your study. If you are not sure if a list item applies to your research, read the appropriate section before selecting a response.

Materials & experimental systems

n/a	Involved in the study	
☐	☒	Antibodies
☒	☐	Eukaryotic cell lines
☒	☐	Palaeontology and archaeology
☒	☐	Animals and other organisms
☐	☒	Human research participants
☒	☐	Clinical data
☒	☐	Dual use research of concern

Methods

n/a	Involved in the study	
☒	☐	ChIP-seq
☐	☒	Flow cytometry
☒	☐	MRI-based neuroimaging

Antibodies

Antibodies used

Surface Marker Fluorophore Clone Final Dilution
Whole blood panel (all from BD Biosciences)
CD14 BV786 M5E2 1:50 (563698)
CD11b BUV805 ICRF44 1:100 (742004)
CD45 BV711 HI30 1:100 (564358)
CD56 BUV737 NCAM16.2 1:100 (612767)
CD11c PE-Cy7 B-ly6 1:100 (561356)
CD63 A647 H5C6 1:100 (561983)
CD4 A700 RPA-T4 1:100 (557922)
CD3 BB515 UCHTI 1:100 (564465)
PD1 BB700 EH12.1 1:100 (566461)
CD15 PE-CF594 W6D3 1:200 (562372)
HLADR V500 G46-6 1:200 (561224)
CD19 BV605 5J25C1 1:200 (562653)
CD8 BV650 RPA-T8 1:200 (563822)
CD16 BUV395 3G8 1:400 (563785)
PBMC panel (all from BD Biosciences)
CD25 PE M-A251 1:25 (555432)
CD127 V450 HIL7RM21 1:50 (560823)
CD3 APCH7 SK7 1:50 (641406)
CD14 BV786 M5E2 1:50 (563698)
CD45RA BV711 HI100 1:100 (563733)
HLADR BB515 G46-6 1:100 (564516)
CD56 BUV737 NCAM16.2 1:100 (612767)
CD11c PE-Cy7 B-ly6 1:100 (561356)
CD4 A700 RPA-T4 1:100 (557922)
PD1 BB700 EH12.1 1:100 (566461)
CCR7 PE-CF594 150503 1:200 (562381)
CD19 BV605 5J25C1 1:200 (562653))

	CD8 BV650 RPA-T8 1:200 (563822)<br>CD16 BUV395 3G8 1:400 (563785)
Validation	Each lot of conjugated antibody is quality control tested by flow cytometry analysis of stained cells using the appropriate positive and negative cell staining and/or activation controls (see supplemental figure 1). All antibodies are mouse anti-human, and were tested on human cells.

Human research participants

Policy information about studies involving human research participants

Population characteristics	Blood samples from children (total 48); 27 SARS-CoV-2 positive (11 acute (4 males), 16 convalescent (9 males)) and 21 SARS-CoV-2 exposed (7 acute (2 males), 14 convalescent (6 males)). Blood samples from adults (total 70): 25 SARS-CoV-2 positive (17 acute (9 males), 8 convalescent (3 males)) and 45 SARS-CoV-2 exposed (21 acute (9 males), 24 convalescent (14 males)). Children ranged from 1-17 years old, and Adults ranged from 19-62 years old. Demographic information is specified in Table 1 of the manuscript.
Recruitment	Patients were recruited through the Respiratory Infection Clinic at the Royal Children's Hospital in March 2020. Criteria for recruitment was a positive COVID-19 case in the household.
Ethics oversight	This project received ethical approval from The Royal Children's Hospital Melbourne Human Research Ethics Committee (HREC): HREC/63666/RCHM-2019. All donors or their legal guardians provided written informed consent.

Note that full information on the approval of the study protocol must also be provided in the manuscript.

Flow Cytometry

Plots

Confirm that:

☒ The axis labels state the marker and fluorochrome used (e.g. CD4-FITC).

☒ The axis scales are clearly visible. Include numbers along axes only for bottom left plot of group (a 'group' is an analysis of identical markers).

☒ All plots are contour plots with outliers or pseudocolor plots.

☒ A numerical value for number of cells or percentage (with statistics) is provided.

Methodology

Sample preparation	Blood was collected in EDTA tubes from each participant at day 12, 37 and 88. Immediately following collection, 100µl of whole blood was aliquoted for flow cytometry analysis. The remaining EDTA blood samples were processed into plasma and PBMC as previously described 28. For flow cytometry analysis of whole blood samples, whole blood was lysed with 1mL of red cell lysis buffer for 10 minutes at room temperature. Cells were washed with 1mL PBS and centrifuged at 350 x g for 5 minutes. Following two more washes, cells were resuspended in PBS for viability staining using near infra-red viability dye according to manufacturers instructions. For flow cytometry analysis of freshly isolated PBMC, cells were washed in 1mL PBS prior to viability staining using BV510 viability dye according to manufacturers instructions. For both whole blood and PBMC samples, the viability dye reaction was stopped by the addition of FACS buffer (2% heat-inactivated FCS in 2mM EDTA) and cells were centrifuged at 350 x g for 5 minutes. Cells were then resuspended in human FC-block according to manufacturers instructions for 5 minutes at room temperature. The whole blood or PBMC antibody cocktails (Extended data Table 1) made up at 2X concentration were added 1:1 with the cells and incubated for 30 minutes on ice. Following staining, cells were washed with 2mL FACS buffer and centrifuged at 350 x g for 5 minutes. Cells were then resuspended in 2% PFA for a 20 minute fixation on ice, washed, and resuspended in 150µl FACS buffer for acquisition using the BD LSR X-20 Fortessa. For all flow cytometry experiments, compensation was performed at the time of sample acquisition using compensation beads.
Instrument	BD Fortessa X-20 (special order research product)
Software	Data collection: DIVA<br>Data analysis: FlowJo V10
Cell population abundance	No cell populations were sorted.
Gating strategy	Within the PBMC fraction, B cells were selected based on CD19 expression, and the total T cell fraction based on CD3 expression. CD4 and CD8 T cells, and their naïve, effector, memory and regulatory (Treg) subsets were also quantified. HLADR + and PD1+ T cells were investigated. CD3-CD19- cells were classified into NK cells (CD56+) and innate cells (HLA-DR+). Within the innate cell fraction, CD14+ monocytes and CD11c+ DCs were identified. Monocyte and NK cell subsets were identified based on CD16 expression. Low density neutrophils were observed in the PBMC fraction at day 88 only, characterised by a high SSC profile, CD16, CD14 and CD11c expression. For whole blood, granulocytes were selected within CD45+ leukocytes based on their SSC profile and CD15 expression. Neutrophils were CD15+CD16+ and eosinophils were CD15+CD16-. This is shown in detail in Extended Data Figure 1.

☒ Tick this box to confirm that a figure exemplifying the gating strategy is provided in the Supplementary Information.
